# Strategies for Extending Metabolomics Studies with Stable Isotope Labelling and Fluxomics

**DOI:** 10.3390/metabo6040032

**Published:** 2016-10-01

**Authors:** Anubhav Srivastava, Greg M. Kowalski, Damien L. Callahan, Peter J. Meikle, Darren J. Creek

**Affiliations:** 1 Monash Institute of Pharmaceutical Sciences, Monash University, Parkville 3052, Melbourne, Victoria, Australia; Darren.Creek@monash.edu; 2 Institute for Physical Activity and Nutrition Research, School of Exercise and Nutrition Sciences, Deakin University, Burwood 3125, Victoria, Australia; greg.kowalski@deakin.edu.au; 3 Centre for Chemistry and Biotechnology, School of Life and Environmental Sciences, Deakin University, Burwood 3125, Victoria, Australia; damien.callahan@deakin.edu.au; 4 Baker IDI Heart and Diabetes Institute, Melbourne 3004, Victoria, Australia; peter.meikle@bakeridi.edu.au

**Keywords:** stable-isotope labelling, metabolomics, fluxomics

## Abstract

This is a perspective from the peer session on stable isotope labelling and fluxomics at the Australian & New Zealand Metabolomics Conference (ANZMET) held from 30 March to 1 April 2016 at La Trobe University, Melbourne, Australia. This report summarizes the key points raised in the peer session which focused on the advantages of using stable isotopes in modern metabolomics and the challenges in conducting flux analyses. The session highlighted the utility of stable isotope labelling in generating reference standards for metabolite identification, absolute quantification, and in the measurement of the dynamic activity of metabolic pathways. The advantages and disadvantages of different approaches of fluxomics analyses including flux balance analysis, metabolic flux analysis and kinetic flux profiling were also discussed along with the use of stable isotope labelling in *in vivo* dynamic metabolomics. A number of crucial technical considerations for designing experiments and analyzing data with stable isotope labelling were discussed which included replication, instrumentation, methods of labelling, tracer dilution and data analysis. This report reflects the current viewpoint on the use of stable isotope labelling in metabolomics experiments, identifying it as a great tool with the potential to improve biological interpretation of metabolomics data in a number of ways.

## 1. Metabolomics

In the postgenomic era, metabolomics has emerged as a powerful approach for enabling the study of the intricate biochemistry of cells, organisms, and systems in response to different conditions such as stress, disease, or nutrition. This detailed analysis of metabolite profiles can provide functionally relevant biochemical information about biological processes. It forms one of the pillars of systems biology, which advocates the need for a holistic view for understanding biological systems. Metabolomics relies on identification and measurement of small molecule metabolites in a biological system, and is usually driven by global data acquisition which leads to hypothesis generation for further research. This approach is typically built on readouts of steady-state metabolite levels/concentrations and thus only presents a snapshot of biological activity. While useful in some circumstances, for example, in detecting in-born errors of metabolism, measurement of static metabolite levels is unable to directly determine the activity of metabolic pathways, which are intrinsically dynamic in nature and often experience minimal metabolite (reactant and product) concentration changes [1,2]. In contrast, unlike static concentration measurements, the use of isotopically labelled metabolic tracers during experimentation adds the dimension of time, thus allowing metabolic pathway kinetics (fluxes) to be measured [3,4,5]. Historically, isotopic tracers (both radioactive and stable) have been at the forefront of fundamental discoveries in biochemistry and physiology and their use is considered the “gold standard” for measuring cell and whole body metabolism [6].

Combining the use of stable isotope labelled metabolic precursors (tracers), and modern analytical techniques (such as high resolution chromatography and mass spectrometry) for separation and detection of chemical species permits characterization of the metabolism of a broad range of biological systems. This can include direct and accurate measurement of turnover of a specific metabolite or even determining entire pathway fluxes *in vitro* or *in vivo* [4,5,7]. A targeted analysis using this approach can quantify the level of activity in specific metabolic pathways and has recently led to a number of important discoveries in cancer biology [8,9], parasitology [10,11], disease biomarkers [12] and plant biology [13] amongst numerous other fields. Moreover, robust untargeted metabolome characterization, leading to pathway mapping throughout the metabolic network has been made possible by combining advanced stable isotope labelling methods with improved genome annotations [14,15,16].

## 2. Stable Isotope Labelling

Stable isotope labelling is becoming increasingly popular in the field of metabolomics for a number of reasons. Being non-hazardous, stable isotopes clearly have a marked advantage over traditionally-used radioisotopes and have almost completely replaced the use of radionuclides in mass spectrometry and NMR based modern metabolomics. The discussion during the peer session encompassed the following broad areas.

### 2.1. Reference Standards

Stable isotope labelling can be used for generating reference standards, which can aid in metabolite identification and absolute quantification by stable isotope dilution strategies. Confident identification and accurate quantification are major bottlenecks in global discovery-based experiments owing to problematic matrix effects which contaminate spectra and suppress signal responses. Stable isotope dilution is the gold standard for accurate quantification and stable isotope reference standards are becoming increasingly available. However, for metabolome-wide approaches the use of individual stable isotope standards is neither practical nor economical. An attractive strategy is to apply labelled standards in a metabolome-wide approach by mixing the experimental biological sample extract with a known biological extract grown in the presence of an exclusively labelled single carbon source and analyzing the resulting isotope labelling patterns, thus permitting precise, relative quantification (corrected for matrix effects and response factors) of many hundreds of metabolites simultaneously [17,18,19,20]. This approach is likely to gain more favor as globally labelled biological samples become more readily available. A modification of the isotope dilution quantification technique can also be used in a targeted manner for absolute quantification of a subset of metabolites. This is achieved by obtaining a linear concentration curve from an unlabelled standard mix of metabolites of interest which contains a predetermined amount of labelled biological extract (also used to spike the samples to be studied). The ratio of unlabelled to labelled standards can then be used to quantify the corresponding metabolites in the experimental samples.

### 2.2. Studying Metabolic Fate of Precursors

The major interest in stable isotope labelling for metabolomics stems from its ability to aid in the measurement of dynamic activity of metabolic pathways, in order to provide mechanistic explanations for the perturbations in steady-state metabolite levels observed in classical metabolomics studies. Stable isotope labelled precursors with uniform or positional labelling of constituent atoms have been used to generate biological samples that allow the study of their metabolic fate throughout the network in an untargeted fashion [14,15,16]. Similar tracer analyses have also been used for many years alongside metabolomics studies to study specific metabolic pathways of interest in a targeted manner [6,21,22]. In addition to the exploration of endogenous pathways, the fate of such tracers has also been exploited for detection of biotransformation products of xenobiotics [23] and to study drug metabolism [24].

### 2.3. Fluxomics Analyses

Fluxomics, in theory refers to the measurement of all the metabolic reaction rates in a biological system, including the directionality of metabolic conversions and import/efflux of metabolites. Like metabolomics, fluxomics requires identification and analysis of metabolites, and in practice, is often limited to known central metabolic pathways involving a subset of metabolites due to an inability to actually measure the entire biological “metabolome” using any single analytical platform. A number of approaches for fluxomics analyses were discussed in the peer session.

#### 2.3.1. Flux Balance Analysis

Owing to the highly dynamic nature of any biological system at a given time, it is difficult to directly measure the metabolic flux. An indirect measurement however can be obtained using the concept of metabolite balancing or flux balance analysis [25]. This traditional approach, also known as stoichiometric metabolic flux analysis [26] is based on the relationships between metabolic substrates and products in the network of predicted biochemical reactions where extracellular metabolites are measured, permitting the measurement of overall system flux. These results are then used to deduce intracellular metabolic fluxes using a stoichiometric matrix which connects intracellular and extracellular metabolites [27]. While flux balance analysis is quite an attractive approach due to its relatively simplistic nature (no tracer experiments are required), its accuracy is completely dependent on the objective function and constraints (assumptions) used to build the model. The choice of objective function is critical to the success of this model, and whilst many models focus on “biomass production”, this is often an inaccurate reflection of the objective of many organisms that must adapt to survive in specific environments. Also, extensive optimization of constraints may be required to evaluate the variability in fluxes due to the dynamic nature of biological systems and resultant fluxes need to be compared to one another and experimental data to assess the suitability of the model for a particular study [28].

Therefore, for successful application of this approach, a thorough understanding of the biological system being studied is critical in order to accurately reconstruct the metabolic network [29]. This method is particularly vulnerable to errors when parameters such as reversible reactions, futile metabolic cycling, and parallel pathways are not accounted for. Furthermore, the use of this method becomes particularly difficult in more complex eukaryotic systems due to numerous cellular compartments (organelles) [26,30]. Moreover, when metabolism is compartmentalized to different organelles, measuring the absolute rate of a certain reaction can be tricky [31]. In these organisms, metabolites can present themselves at more than one location in the cell, mostly due to the presence of multiple organelle specific isoforms of corresponding enzymes and a requirement for maintenance of certain metabolite gradients in different organelles necessary for physiological equilibrium of the cells. Flux balance analyses have to utilize additional specific metabolic constraints corresponding to organelle restrictions for predicted localized reactions, which is difficult to calculate for most organisms. Nevertheless, flux balance analysis is widely used in prokaryotic systems and has been applied for predictions of metabolic fluxes in some eukaryotes [32,33,34]. However, the method has no practical application for *in vivo* studies in animals or humans, owing to a much higher degree of complexity which results from an interconnection of different cell and organ systems that simultaneously produce, transfer, and consume multiple substrates.

#### 2.3.2. Metabolic Flux Analysis

Flux balance analysis can be improved by implementing a method called metabolic flux analysis and using stable isotope labelling to fill in the gaps in data that arise due to compartmentalization or cycling of metabolites. This can be achieved by using a number of stable isotopes of atoms which occur naturally in biomolecules (such as ^2^H, ^13^C, ^15^N, and ^34^S) and performing labelling experiments over a time-course to observe the dynamic nature of metabolism in a variety of biological systems including unicellular pathogens, plants, and mammalian cells [30,35,36,37,38]. The experimental design for metabolic flux analysis requires long term exposure of cells to labelled precursors, allowing equilibration of label incorporation within the metabolic network. The labelling pattern of downstream metabolites resulting from the labelled precursor is then analyzed in order to measure the proportion of flux at different nodes in the metabolic pathway. Prior knowledge of anticipated pathways in different organelles and fate of carbon molecules (in case of labelled glucose or glutamine precursors) [10,22] is required to deduce the information about relative activity of metabolic branches. For example, the arrangement of carbon backbones in multiple labelled precursors such as ^13^C-glucose and ^13^C-glutamine, as they are metabolized in a cascade of enzymatic reactions can be exploited to undertake isotopomer analysis in order to pinpoint specific adaptations in central carbon metabolism [11].

Metabolic flux analysis is widely used for studying central carbon metabolism and a number of pathways which utilize carbon substrates can be explored in a relatively simple experiment. The minimum required time for exposure of cells to a ^13^C labelled precursor in these experiments depends on the time required to reach isotopic steady state. Attainment of an isotopic steady state ensures that the relative fractions of metabolite isotopomers representing the labelling pattern are constant over time and can be assessed easily [39]. This varies for different intermediates in a metabolic network and is controlled by their distance from the entry point of the labelled precursor in the pathway and is also controlled by the rate of reaction and metabolite pool size. For example, labelled glucose can label glycolytic intermediates within seconds to minutes; however, acetate labelling can take many hours [5,16,40].

A variation of this approach called isotopically nonstationary metabolic flux analysis (INST-MFA) has been used to analyze mass isotopomer distributions of metabolites based on their atomic rearrangements in autotrophs. These organisms are sampled before achieving isotopic steady state following ^13^CO_2_ treatment. This approach is able to distinguish contributions from different metabolic pathways, providing higher flux resolution without direct quantification of metabolites [41,42]. This approach has an advantage over kinetic flux profiling (see later) which uses a differential equation to model the isotopic dilution of a pool of one isotopomer. As INST-MFA models all isotopomers, it is more accurate when bond-breaking reactions are part of metabolism [43,44].

#### 2.3.3. Kinetic Flux Profiling

Kinetic flux profiling is an approach which can provide the most dynamic information in a metabolomics experiment, but requires extensive modelling and specialized experimental methodology. It uses dynamic isotopomer distribution data in order to estimate absolute flux along metabolic pathways. Typically, this approach assumes that the magnitude of measured metabolic flux is proportional to the rate of transmission of labelled isotopes from the precursor to subsequent products. It considers steady state dynamics for influx and efflux of a given metabolite and the sample preparation involves switching from an unlabelled precursor to an isotopically labelled precursor at a given time. These assumed steady state dynamics are true for cells undergoing rapid proliferation, provided nutrient resources are not limiting. Under these conditions, when a precursor is switched from an unlabelled to labelled form, the immediate product of the precursor is replaced by its labelled form. Measurement of concentrations of the labelled forms over time—as the label gradually infiltrates the metabolite pool—can be used to estimate the influx and efflux [45]. Kinetic flux profiling has been used to compare fluxes between different experimental conditions by performing relative quantitation, leading to important biological discoveries, e.g., resolution of a branched tricarboxylic acid cycle in bacteria [46], establishment of two independent acetyl-CoA synthesis pathways in the malaria parasite [47] and understanding nitrogen utilization pathways in green algae [48].

#### 2.3.4. *In Vivo* Dynamic Metabolomics and Stable Isotope Labelling Studies

While metabolic flux analysis has been extensively used in microbial, plant, and—more recently—mammalian cell culture systems [30,38], its major limitation lies in its lack of application to *in vivo* animal and human experimentation. Due to the unpredictable nature of a multi-organ system that is shared by the one continually recirculating blood supply, it is difficult, perhaps even impossible, to model (“constrain”) the entire metabolism of an organ (or organs) in an intact animal or human. For example, when an isotopic tracer is administered (i.e., infused or ingested) in animals or humans, the tracer can be utilized by one organ where it is metabolized and recycled to another form and released into the circulation, ready to be taken up and recycled again by another organ. This can occur many times, thus resulting in the formation of a range of secondary tracers (metabolites) with “scrambled” labelling patterns when compared to the originally administered tracer [6]. However, despite these complications, metabolomics-based stable isotope labelling has been used *in vivo* to provide both qualitative and quantitative information on the fate of substrates across multiple metabolic pathways in different organs. By administering stable isotope labelled metabolic precursors, then rapidly harvesting animal organs, or collecting surgical biopsy/resection specimens from humans, targeted metabolomics analyses have been used to determine the contribution that specific substrates make to pathways of central carbon metabolism in both healthy and diseased states [8,49,50,51,52,53,54,55,56,57]. The application of stable isotopes combined with metabolomic analytical strategies will no doubt be a growing area in the future and will hopefully provide a greater understanding of metabolism and metabolic regulation under physiological conditions.

## 3. Technical Considerations

In this session, peers also discussed the importance of a number of technical issues which should be addressed early to ensure reproducibility and acquisition of high quality metabolomics data for stable isotope labelling experiments. Some of these are discussed below.

### 3.1. Importance of Biological and Technical Replicates

Any good experimental design incorporates a plan for replication of the experimental conditions and the power of the study is heavily dependent on the reproducibility of the data obtained, irrespective of the approach. Replicates are essential as they provide multiple measurement points for any given metabolite in a sample, and averaging across replicates improves accuracy and data resolution. Furthermore, replication provides a means of identifying anomalies in the experimental procedure as outliers and, most importantly, they provide measurements of the variability in experimental data that are used for determination of significant differences between study factors. As metabolomics experiments involve multiple steps between sample preparation and analysis, the probability of user-induced variation increases with every step. Usually, with validated procedures and good laboratory practice, technical variability can be kept to the minimum and the balance of the resources (time/biological material) should be tipped towards obtaining biological replicates. However, due to the nature of analytical methods used in metabolomics, it may be desirable to capture technical variability either by obtaining technical replicates, or by the addition of batch-wise quality control samples [58].

If internal standardization is achieved in an identical manner for all variables under testing (time-points, drug treatments etc.) in stable isotope labelled metabolomics experiments, the technical variance is negligible compared to label-free comparative metabolomics studies as the isotopologue peaks are detected in the same sample as the unlabelled (or other isotopologue) peaks. For this reason, fewer technical replicates can be justified in order to offset the increased costs associated with stable isotopes. Nevertheless, intrinsic biological variance remains in even the most tightly controlled experiments, and some degree of biological replication remains essential in order to obtain statistical significance.

### 3.2. Key Instrument Considerations

Only MS instruments with resolving powers above 100,000 are able to resolve most co-eluting isobaric metabolite mass signals [59]. However, metabolomics using stable isotope labelling does not necessarily require high resolution instrumentation, provided the chromatographic selectivity is high enough to keep isobaric metabolites (and isotopes peaks from other metabolites) from overlapping. For example, GC separations are often suitable for combination with cheaper, unit resolution (single quadrupole) MS. On the other hand, the high selectivity separations (e.g., GC) can result in a shift in the retention time of isotopologues which are expected to co-elute. This effect is more pronounced in deuterium (^2^H) labelling but is still apparent for ^13^C and therefore this shift must be taken into account in data processing. Retention time shifts due to ^13^C incorporation are insignificant for small molecules, for example TCA cycle intermediates, however a small shift may be observed for larger molecules, such as fatty acids, when using a highly selective GC method and therefore the data processing method must ensure peak areas for each isotopologue are measured accurately [60].

The linear dynamic range of a method is an important consideration while developing quantitative analyses, however, it is often overlooked when adapting methods to different instruments. Increasing sensitivity of MS instrumentation is not necessarily associated with an increase in linear dynamic range. Most modern MS instruments report a within-scan linear dynamic range of 4–5 orders of magnitude, which does not cover the full concentration range of metabolites and isotopologues likely to be found in a complex metabolomics sample. Therefore, sample loading and/or analytical methods must be adapted to ensure that the instrument can measure isotope profiles within its linear range. Without this consideration, large errors in the measurement of isotopologue ratios can occur and the inclusion of standard curves is therefore necessary to validate the linear dynamic range of a method.

### 3.3. Procedures for Addition of Stable Isotope Precursors to Experimental Systems

The procedures for addition of stable isotope precursors must be considered in the context of the biological system under study and the aim of the experiment. In many cases, for metabolic flux analysis and generation of reference standards, an isotopic steady state is desired. The time taken to reach isotopic steady state is dependent on the metabolic flux within the pathways of interest under the specific experimental conditions. Often a pilot study is necessary to determine an appropriate experiment duration.

In the case of kinetic flux profiling experiments, a series of samples are generally obtained shortly after addition of the labelled precursor. Meaningful interpretation of these studies require the system to be at metabolic steady state throughout the experiment, which necessitates careful consideration of the method used to introduce the stable isotope labelled precursor. Media exchange techniques that rely on centrifugation or filtration, or washing protocols that utilize nutrient-free media, are likely to significantly perturb metabolism and influence the resulting flux measurements.

### 3.4. Stable Isotope Labelled Nutrient Selection and Tracer Dilution

The choice of stable isotope labelled nutrient is a critical first step in these studies, and is highly dependent on the research questions [5,61,62]. For metabolome-wide stable isotope labelling studies, a uniformly ^13^C labelled central carbon source such as glucose (or CO_2_ in plants if a time course study is desired) can be highly informative. However, it is common to perform labelling studies subsequent to the initial hypothesis-generating metabolomics study, and labelled nutrients can be selected based on the published or predicted precursor(s) for the metabolic pathway(s) of interest [24].

A critical point that was raised in the peer session discussion was the importance of accounting for tracer dilution from unlabelled precursors. It was noted that even when care is taken to account for any pre-existing unlabelled precursor at the start of such studies, such forms may further originate from recycling of other metabolic intermediates or environmental fixation and may dilute the labelled tracer along the path of synthesis of downstream products. To overcome this issue, determination of precursor dilution by unlabelled metabolites is necessary, and this can often be achieved by quantitative mass isotopomer distribution analysis [63,64,65].

In some cases, it is informative to study the metabolism of partly-labelled tracer mixtures, due to the complex architecture of metabolic pathways. For example, a 50% labelled/unlabelled mixture of U-^13^C-glucose allows differentiation of the flux in each of the two branches (oxidative vs. non-oxidative) of the pentose phosphate pathway (PPP) in a highly glycolytic cell, which would not be possible from a 100% U-^13^C-glucose precursor (which would completely label all PPP intermediates at isotopic steady state) [14,16]. Non-uniformly labelled tracers are also useful for specific applications, such as the use of 1, 2-^13^C-glucose for differentiating glycolytic and PPP flux.

### 3.5. Correction for Abundance of Naturally Occurring Isotopes and Other Background

Due to the presence of naturally occurring isotopes of metabolites, background subtraction is required prior to isotopologue data interpretation [5,66,67,68]. This is more of an issue for low resolution mass-spectrometers where discrete isotopically-resolved isotopologues (such as those resulting from different isotopes e.g., ^2^H and ^13^C) cannot be differentiated from one another. This isobaric background signal can be minimized by using ultra-high resolution mass spectrometers for data acquisition and post-processing computation which incorporates background correction [68,69]. Background contamination of samples can also result in underestimation of labelling. Fatty acids, for example are particularly challenging due to their use in the manufacture of plastic tubes used for metabolite extractions [70]. In some cases, the background from tubes may not be consistent from batch to batch of plasticware, further complicating background subtrations. Furthermore, the addition of derivatization agents for GC-MS studies leads to higher molecular weight molecules, and therefore higher natural isotope abundance than the underivatised metabolites, with this higher background being particularly evident with silylating agents [68]. These factors need to be considered both at the experiment design stage and during data processing.

### 3.6. Alternative Uses of Stable Isotopes to Complement Metabolomics Studies

In addition to administration of isotopically labelled precursor molecules (such as ^13^C glucose, amino acids, acetate, etc.) and observing their metabolic incorporation and/or dilution in biological systems, it is also possible to use other labelling approaches combined with mass spectrometric and/or NMR analysis to understand cellular metabolism and growth characteristics. For example, the use of deuterated “heavy” water (^2^H_2_O) has proven to be a particularly powerful approach for measuring the synthesis rates of proteins [71,72,73,74,75], lipids (*de novo* lipogenesis, esterification, and chain elongation) [67,76,77,78], DNA [79,80], and RNA [81], thus providing information on biomass synthesis and cell division rates, both *in vivo* and *in vitro*. Furthermore, ^2^H_2_O labelling can be used to quantify the pathways used to produce glucose and glycogen, thus providing a way to measure gluconeogenic and glycogenolytic/glycogenic pathway activity [82,83,84,85,86,87]. The basis of performing ^2^H_2_O labelling lies in the fact that many different biomolecules incorporate hydrogen atoms (protons) from body/cellular water via enzyme catalyzed reactions [88,89,90]. Therefore, upon the administration of low quantities of ^2^H_2_O into a living system, deuterium (^2^H) atoms become stably incorporated into C-H bonds of newly synthesized biomolecules, essentially “tagging” them. The degree to which molecules (particularly macromolecules) become enriched with ^2^H atoms depends on both their rates of synthesis and the pathways used to synthesize them. ^2^H_2_O can be administered to animals or humans in drinking water, or directly into the cell culture media *in vitro*, which results in the enrichment of the body/cellular water pool. A major advantage of using ^2^H_2_O is that it can be used to probe multiple metabolic processes simultaneously and can be easily and safely administered for long periods of time *in vivo* and *in vitro*, permitting flux analysis in free living conditions or long term culture experiments [3,73,79,91]. However, it should be noted that high levels of ^2^H_2_O enrichment can have toxic effects on cellular systems due to the slightly stronger bonds that deuterium forms with other atoms when compared to hydrogen, thus creating kinetic isotope effects on chemical reactions [92,93]. The toxic effects can be largely avoided by maintaining ^2^H_2_O enrichments of body water/cell culture media to <10%, although the kinetic isotope effect should be considered when modelling and interpreting data from ^2^H_2_O labelling [92,93]. ^2^H_2_O has also recently been combined with other “omics” technologies including proteomics [74,75,94,95,96], permitting the determination of synthetic rates of individual proteins on a global level *in vivo*, and imaging mass spectrometry [97], permitting the imaging of newly synthesized lipids in tissue histology samples, termed “kinetic histochemistry”. Thus, in addition to using metabolomics and substrate specific tracers for mapping metabolic pathway activity, ^2^H_2_O labelling can provide complementary information on the synthetic rates of numerous cellular components and their biosynthetic pathways (i.e., gluconeogenesis vs. glycogenolysis; *de novo* lipogenesis vs. preformed fatty acid esterification).

## 4. Conclusions

Whether being used as a hypothesis-generating holistic approach, or for hypothesis-driven analysis of specific pathways, stable isotope labelling is a powerful tool that can enhance the biological interpretation of metabolomics data. Stable isotope labelling can help alleviate a number of issues associated with metabolomics data analysis and provide confidence in assigning correct identification to metabolite features. Quantification of metabolites in real time using stable isotope labelling allows accurate measurements for fluxomics studies. For most biological experiments investigating mechanisms of metabolic perturbation, targeted tracer studies or metabolic flux analysis are the preferred methods, due to ease of experimental design and data analysis. These approaches often reveal sufficient information about the metabolic pathways of interest to interpret observed biological phenomena without the complexity associated with detailed kinetic flux analysis.

Thanks to stable isotope labelling, we are increasingly advancing from discovery-based global metabolomics approaches and becoming capable of teasing out mechanisms of interplay of metabolic pathways in intricate networks. We hope that the use of stable isotope labelling will become routine in metabolomics experiments in the future and will continue to play a major role in biochemical elucidation of existing pathways along with identification of novel metabolic networks.
